# Innovative Approaches to Continuing Medical Education

**DOI:** 10.7759/cureus.74638

**Published:** 2024-11-27

**Authors:** Muhammad Mannan, Asra Afzal, Usman Hafeez, Rizwan Akbar, Rimsha Tahir

**Affiliations:** 1 Trauma and Orthopaedics, Sheikh Zayed Medical College and Hospital, Rahim Yar Khan, PAK; 2 Orthopaedic Surgery, University Hospitals Birmingham NHS Foundation Trust, Birmingham, GBR; 3 Obstetrics and Gynaecology, University Hospitals Birmingham NHS Foundation Trust, Birmingham, GBR; 4 Trauma and Orthopaedics, University Hospitals Birmingham NHS Foundation Trust, Birmingham, GBR; 5 Trauma and Orthopaedics, Birmingham Heartlands Hospital, Birmingham, GBR; 6 Trauma and Orthopaedics, Hayatabad Medical Complex Peshawar, Peshawar, PAK

**Keywords:** continuing medical education (cme), digital platforms in healthcare, healthcare professional development, just-in-time training (jitt), microlearning

## Abstract

Background: Continuing medical education (CME) is vital for healthcare professionals to maintain clinical competence and keep pace with the rapidly evolving medical landscape. Traditional CME models often fall short of meeting the dynamic needs of modern healthcare professionals.

Objective: This study explores innovative CME methods, including microlearning, Just-in-Time Training (JITT), and digital platforms, to assess their effectiveness and identify potential barriers to adoption.

Methods: A mixed-methods approach was employed, integrating a systematic literature review with quantitative surveys of 300 healthcare professionals and qualitative interviews with 50 CME administrators. The study was conducted at Birmingham Heartlands Hospital. Statistical analyses were performed on the quantitative data, while thematic analysis was applied to qualitative responses to identify key themes and insights.

Results: Microlearning was regularly utilized by 234 participants (78%), with 204 participants (68%) indicating it as their preferred learning method due to its flexibility and efficiency. JITT was particularly valued in critical care settings, with 135 participants (45%) employing it and 210 participants (70%) expressing high satisfaction. Digital platforms were accessed by 276 participants (92%), with 264 participants (88%) expressing satisfaction with their content diversity and ease of access. Major barriers identified included digital literacy challenges, reported by 156 participants (52%), and technological access limitations, reported by 144 participants (48%).

Conclusion: Innovative CME approaches like microlearning, JITT, and digital platforms show great promise in aligning with the current needs of healthcare professionals. Addressing barriers such as digital literacy and technological infrastructure is essential to ensure widespread adoption and maximize their impact on healthcare outcomes.

## Introduction

Continuous professional development is essential in modern healthcare, ensuring that clinicians remain current with the latest advancements and maintain the skills needed to deliver high-quality patient care [1]. Continuing medical education (CME) is crucial in updating healthcare professionals on emerging clinical guidelines, evolving treatment protocols, and new medical technologies [2]. While traditional CME methods, such as in-person conferences and workshops, have long been the standard, they face several limitations in today's dynamic healthcare landscape [3].

Traditional CME often struggles to adapt to the fast-paced nature of medical practice. Several factors contribute to its shortcomings, including inflexible scheduling, as conventional CME events occur at set times and locations that may not align with the busy and unpredictable schedules of healthcare professionals [4]. High costs associated with travel, accommodation, and event fees can also be prohibitive, particularly for clinicians working in resource-limited settings [5]. Additionally, geographical barriers pose significant challenges for those in remote or underserved areas, making it difficult to access in-person CME opportunities [6]. Furthermore, traditional lecture-based approaches often rely on passive learning methods, which may not actively engage participants or facilitate long-term knowledge retention [7].

Given these limitations, there is a growing interest in more flexible, technology-driven CME approaches. Methods such as microlearning, Just-in-Time Training (JITT), and digital platforms have emerged as promising alternatives, providing personalized, accessible, and interactive learning experiences [8].

Microlearning delivers educational content through concise, focused modules that enhance knowledge retention by encouraging active engagement and utilizing spaced repetition techniques. For example, a healthcare professional might complete a five-minute module on recognizing early symptoms of sepsis, allowing them to review essential signs and symptoms quickly between patient consultations. Additionally, modules on surgical protocols or medication guidelines can be accessed just before specific tasks, reinforcing key steps and ensuring best practices. This format is ideal for high-demand environments, enabling learning that fits seamlessly into short, available time slots [9]. Its adaptable format allows healthcare professionals to engage in quick, on-the-go learning that fits seamlessly within their busy schedules [10].

JITT provides immediate, context-specific information directly at the point of care, aiding in real-time clinical decision-making [11]. This method is particularly valuable in critical settings like emergency departments and intensive care units (ICU), where quick access to relevant information can impact patient outcomes significantly. For example, in an ICU, a physician might use JITT to review emergency protocols for respiratory failure just before attending to a critical case, ensuring they have the latest guidelines in mind [12].

Digital platforms also play a key role in this approach by offering a variety of online tools, including e-learning modules, webinars, mobile apps, and virtual simulations [13]. These tools support interactive, self-paced learning, allowing professionals to access content that meets their unique learning needs. For instance, a clinician could use a mobile app to watch a brief instructional video on a new surgical technique before a procedure or participate in a simulation that allows practice in a safe, virtual environment. This flexibility enables healthcare professionals to stay informed and well-prepared, even in high-pressure situations [14,15].

Despite the advantages, digital literacy, defined as the ability to effectively and confidently use digital tools, platforms, and resources, is crucial for healthcare professionals in navigating online training, telemedicine, and electronic health records. Some challenges, like limitations in technology infrastructure, such as unreliable internet connections or outdated devices, can impede access to these essential digital resources [16]. Addressing these issues is essential to ensure healthcare professionals can fully benefit from digital education and resources.

This study aims to evaluate the effectiveness of these emerging approaches and identify the key barriers to their broader implementation.

## Materials and methods

This study utilized a mixed-methods approach, combining quantitative surveys and qualitative interviews to provide a comprehensive evaluation of the usage, effectiveness, and perceived barriers of innovative CME methods.

Data collection

The data collection process involved three components.

Literature Review

Systematic reviews published between 2010 and 2023 were reviewed using databases such as PubMed, MEDLINE, and Google Scholar. Search terms included "Continuing Medical Education", "microlearning", "Just-in-Time Training", and "digital platforms". The review focused on identifying CME innovations, assessing their effectiveness, and understanding the challenges associated with their implementation. Based on information gathered from these systematic reviews, survey and interview questions (Appendix 1 and Appendix 2) were structured.

Surveys

A structured survey was administered to 300 healthcare professionals across various specialties at Birmingham Heartlands Hospital. The survey looks at demographic data, experience, and preferences of healthcare professionals regarding varying methods of CME, including but not limited to microlearning, JITT, and digital and traditional formats. It also investigates how often, how satisfied the participants are, how effective, and what are barriers such as the digital divide and technology access. Participants suggest ways of increasing the relevance, adequacy for the audience, and scope of CME offered and name their priorities in methods used as well as how much further they would want to improve their digital literacy. In addition, the questionnaire is intended to provide useful and constructive criticism towards the effective delivery of CME.

Interviews

Semi-structured interviews were conducted with 50 CME administrators who had participated in a survey and were selected by a short call interview on the basis of their expertise to explore the factors that facilitate or hinder the adoption of innovative CME methods in healthcare settings. This interview guide explores the role of CME administrators, their experience with traditional and innovative methods like microlearning and digital platforms, and their views on the shift toward these approaches. It addresses challenges such as resistance, digital literacy, and technological barriers while evaluating the effectiveness of newer methods in improving skills and knowledge retention. It also seeks insights into content engagement, feedback from healthcare professionals, and strategies for overcoming obstacles. The interview concludes by examining future trends, the role of technology, and recommendations for enhancing CME accessibility and effectiveness.

Data analysis

Quantitative Data

Descriptive statistics were used to summarize survey data, while inferential analyses such as chi-squared tests and logistic regression were applied to explore the relationships between usage patterns, preferences, satisfaction levels, and perceived barriers. Statistical significance was determined at a p-value threshold of 0.05. Data reliability was assessed using Cronbach's alpha, yielding a value of 0.87, indicating high internal consistency.

Qualitative Data

Thematic analysis was conducted on interview responses to identify recurring themes and gain insights into the effectiveness of CME methods and the challenges associated with their implementation. This analysis provided a nuanced understanding of the factors influencing the adoption of innovative CME approaches.

## Results

The findings from the survey provide valuable insights into the usage, preference, and satisfaction levels of various CME methods among healthcare professionals, as well as the statistical significance associated with each method's adoption. Table 1 presents a summary of these findings.

**Table 1:** Usage, preference, satisfaction levels, and statistical significance of CME methods. CME: continuing medical education; JITT: Just-in-Time Training

CME method	Regular use (N, %)	Preferred method (N, %)	High satisfaction (N, %)	Statistical significance
Microlearning	234 (78%)	204 (68%)	246 (82%)	p<0.001
JITT	135 (45%)	120 (40%)	210 (70%)	Not significant
Digital platforms	276 (92%)	180 (60%)	264 (88%)	β=0.65; p<0.01 (ease of access)
				β=0.48; p<0.05 (content variety)

Microlearning emerged as a widely adopted method, with 234 participants (78%) utilizing it regularly. Its flexibility was a significant factor, making it the preferred choice for 204 participants (68%). Additionally, 246 participants (82%) expressed high satisfaction with microlearning, underscoring its effectiveness in enhancing knowledge retention. The statistical analysis revealed a significant preference for microlearning over other CME methods (p<0.001), indicating its strong acceptance and popularity among healthcare professionals.

JITT showed a more moderate level of adoption, being used by 135 participants (45%). It was particularly valued in high-pressure environments such as critical care, where immediate applicability is crucial. Despite 120 participants (40%) identifying JITT as their preferred method and 210 participants (70%) expressing high satisfaction, the statistical analysis did not reveal a significant association for JITT compared to the other CME methods. This suggests that while JITT has its merits and a dedicated user base, it may not be the most preferred approach overall within the surveyed group.

Digital platforms had the highest adoption rate, with 276 participants (92%) regularly accessing these resources. These platforms were favored by 180 participants (60%), who appreciated their variety of content and ease of access. The satisfaction level was equally high, with 264 participants (88%) expressing contentment with their use. Further statistical analysis showed that high satisfaction with digital platforms was significantly linked to two key factors: ease of access (β=0.65; p<0.01) and content variety (β=0.48; p<0.05). These findings emphasize the importance of accessibility and the availability of diverse content in the success of digital platforms as a preferred CME method.

This version provides a clear representation of the survey results by including the number of participants who expressed each response, alongside the percentages. This is presented in Table 1.

Digital literacy challenges were noted by 156 participants (52%), with a higher prevalence among professionals aged 50 and above. This finding underscores the need for targeted digital literacy training to help these professionals effectively navigate and utilize modern CME platforms. Additionally, technological access limitations were reported by 144 participants (48%), particularly those in rural areas. These individuals pointed to disparities in technological infrastructure and the availability of reliable internet access as major obstacles to engaging with digital CME resources. These findings are summarized in Table 2.

**Table 2:** Perceived barriers to the adoption of innovative CME methods. CME: continuing medical education

Barrier	% of respondents (N, %)	Description
Digital literacy challenges	156 (52%)	Reported mostly by professionals aged 50 and above; indicates a need for digital literacy training.
Technological access limitations	144 (48%)	Noted particularly by those in rural areas, pointing to disparities in technological infrastructure and internet access.

Flexibility and accessibility were highlighted by 264 participants (88%) as key advantages of innovative CME methods, which enable the seamless integration of learning into daily routines and accommodate the busy schedules of healthcare professionals.

Enhanced knowledge retention was reported by 225 participants (75%), particularly in relation to microlearning. The concise, targeted nature of microlearning modules was viewed as essential for reinforcing learning and improving information recall during clinical practice.

The ability to support real-time decision-making was recognized as a significant benefit of JITT, with 195 participants (65%) noting its effectiveness, especially in high-pressure environments such as critical care and emergency settings.

Digital literacy barriers were identified by 156 participants (52%), consistent with the findings from the quantitative survey. This underscores the need for digital literacy training programs to facilitate the effective use of online CME platforms.

The importance of content standardization was raised by 150 participants (50%), who emphasized that establishing standardized accreditation processes is crucial to ensure the quality, consistency, and relevance of online CME materials. This is presented in Table 3.

**Table 3:** Key themes identified in qualitative analysis of CME methods. CME: continuing medical education; JITT: Just-in-Time Training

Theme	% of interviewees	N (out of 300)	Description
Flexibility and accessibility	88%	264	Emphasized as a crucial benefit, allowing the integration of learning into daily routines.
Enhanced knowledge retention	75%	225	Recognized particularly in microlearning, aiding in information recall during practice.
Immediate clinical application	65%	195	A strength of JITT, supporting real-time decision-making in critical care and emergency settings.
Digital literacy barriers	52%	156	Echoes quantitative findings; highlights the need for digital literacy programs.

## Discussion

The findings of this study highlight the growing acceptance of innovative CME approaches among healthcare professionals, particularly the use of microlearning and digital platforms. These methods have proven highly adaptable to the fast-paced and demanding nature of modern healthcare, offering flexibility and efficiency that traditional CME models often lack [1,2].

Microlearning, in particular, emerged as a highly effective method, with 246 participants (82%) expressing high satisfaction. Its concise, focused delivery allows healthcare professionals to seamlessly integrate learning into their daily routines without interrupting patient care [8]. The ability to engage in quick, targeted educational activities makes microlearning an efficient way for professionals to update clinical knowledge and acquire new skills, underscoring its role in enhancing knowledge retention [9]. On the other hand, microlearning can oversimplify complex topics, overwhelm learners, and require significant resources for implementation. Its limitations, such as retention issues and reduced interactivity, highlight the need for integration with broader training strategies.

JITT has also demonstrated significant value in critical care and emergency settings, where quick decision-making is essential. With 210 participants (70%) expressing satisfaction, JITT has shown its effectiveness in providing real-time, context-specific information that supports clinical decision-making under pressure [11]. JITT not only improves clinical outcomes but also cultivates a culture of excellence and continuous learning by giving professionals, especially novices, immediate access to vital information in situations where the stakes are frequently life and death [13].

Digital platforms, which provide the most comprehensive array of learning resources, were accessed by 276 participants (92%). These platforms received high satisfaction ratings from 264 participants (88%), who appreciated the variety of content and ease of access. The satisfaction observed associated with digital platforms and factors such as accessibility (β=0.65; p<0.01) and content diversity (β=0.48; p<0.05) in this study were also demonstrated with their effectiveness in catering to a wide range of educational needs by Ellaway and Masters in their e-learning guide [12]. Digital platforms' ability to offer personalized and self-directed learning experiences makes them a vital resource for supporting lifelong learning [14,15].

Despite the benefits of these innovative CME methods, the study identified several barriers to their widespread adoption. Digital literacy challenges were noted by 156 participants (52%), particularly among older professionals, indicating the need for targeted digital literacy training programs [7]. Without adequate training, healthcare workers may struggle to fully leverage these advanced CME tools, potentially widening existing knowledge gaps [5]. Additionally, technological access limitations, reported by 144 participants (48%), particularly in rural or underserved areas, highlight disparities in infrastructure and reliable internet access. Addressing these issues is crucial to ensuring equitable access to CME programs and fostering inclusive participation across various regions [6]. To address barriers to CME adoption, targeted digital literacy training, user-friendly platforms, and mentorship programs must be implemented. Enhancing technological access through infrastructure development, mobile-optimized tools, offline options, and policy advocacy for equitable participation can be an optimal solution.

Limitations

This study has several limitations that should be considered when interpreting the findings. First, the research was conducted at Birmingham Heartlands Hospital, which may limit the generalizability of the results to other healthcare settings or regions with different technological capabilities and CME needs. The reliance on self-reported data through surveys introduces the potential for response bias, as participants may have overstated their use or satisfaction with CME methods. Additionally, the qualitative data, although valuable, could be influenced by interpretation bias during thematic analysis, which may affect the identification of key themes.

Another limitation is the cross-sectional design of the study, which provides a snapshot of CME usage and preferences at a single point in time but does not capture how these patterns may evolve. A longitudinal study could offer more robust insights into the long-term effectiveness and sustainability of these innovative CME methods. Finally, because the study relied heavily on digital platforms, it may have unintentionally excluded healthcare professionals who have limited or no access to such technologies, particularly those in underserved areas. This could lead to an under-representation of the challenges faced by these individuals, skewing the results toward those with better technological resources. Addressing these limitations in future research would offer a more comprehensive understanding of the integration and impact of innovative CME methods across a broader range of healthcare environments.

## Conclusions

Innovative CME approaches, microlearning, JITT, and digital platforms, are effective and adaptable methods that align with the evolving needs of healthcare professionals, with 82% of participants expressing high satisfaction with microlearning due to its concise delivery and seamless integration into daily routines, despite concerns about oversimplification. These methods provide flexibility, enhance knowledge retention, and support real-time clinical decision-making, making them valuable tools in modern medical education. Barriers included digital literacy challenges (52%) and technological access issues (48%), underscoring the need for targeted training, infrastructure development, and inclusive policy initiatives to maximize the benefits of these methods and their widespread adoption.

To overcome these challenges, healthcare organizations should focus on enhancing digital literacy through comprehensive training programs, particularly for those less familiar with digital tools. Additionally, investing in technological infrastructure is essential to provide reliable internet access across all regions, especially underserved areas. Standardizing content with accreditation standards and quality benchmarks will ensure the reliability and relevance of digital CME resources. By implementing these strategies, the healthcare sector can create an environment that fosters lifelong learning, ultimately leading to improved patient care outcomes.

## Appendices

Appendix 1: Survey questions for CME study

Section 1: Demographic Information

1.   What is your age group?

o    Under 30

o    30-39

o    40-49

o    50-59

o    60 and above

2.   What is your current role in the healthcare sector?

o    Physician

o    Nurse

o    Specialist

o    Medical assistant

o    Others (please specify)

3.   How many years of experience do you have in the healthcare field?

o    Less than 5 years

o    5-10 years

o    11-20 years

o    More than 20 years

4.   What specialty or department do you primarily work in?

o    Emergency medicine

o    General medicine

o    Surgery

o    Critical care

o    Others (please specify)

Section 2: Usage of CME Methods

5.  Which of the following CME methods have you used in the past 12 months? (Select all that apply)

o   In-person conferences

o   Webinars

o   Microlearning

o   JITT

o   Digital platforms (e-learning modules, apps, virtual simulations)

6.   How frequently do you use microlearning for CME purposes?

o    Daily

o    Weekly

o    Monthly

o    Rarely

o    Never

7.    How frequently do you use JITT in your practice?

o    Daily

o    Weekly

o    Monthly

o    Rarely

o    Never

8.   How frequently do you access digital platforms for CME?

o    Daily

o    Weekly

o    Monthly

o    Rarely

o    Never

Section 3: Preferences and Satisfaction

9.   Which CME method do you prefer most for updating your clinical knowledge?

o   Microlearning

o   JITT

o   Digital platforms

o   Traditional in-person conferences/workshops

o   Others (please specify)

10.  How satisfied are you with microlearning as a CME method?

o   Very satisfied

o   Satisfied

o   Neutral

o   Unsatisfied

o   Very unsatisfied

11.  How satisfied are you with JITT?

o    Very satisfied

o    Satisfied

o    Neutral

o    Unsatisfied

o    Very unsatisfied

12.  How satisfied are you with digital platforms for CME?

o   Very satisfied

o   Satisfied

o   Neutral

o   Unsatisfied

o   Very unsatisfied

Section 4: Barriers to Adoption

13. What challenges have you faced while using digital CME platforms? (Select all that apply)

o   Difficulty navigating the platforms

o   Lack of time

o   Poor internet connection

o   Limited access to necessary technology/devices

o   Lack of digital literacy

o   No challenges

14.  How would you rate your confidence in using digital tools for learning?

o   Very confident

o   Confident

o   Neutral

o   Not confident

o   Not at all confident

15.  Have you experienced any barriers specifically related to accessing microlearning or JITT resources?

o   Yes (please specify)

o   No

Section 5: Effectiveness and Feedback

16. How effective do you find microlearning in improving your clinical knowledge and skills?

o   Very effective

o   Effective

o   Neutral

o   Ineffective

o   Very ineffective

17.  How effective do you find JITT in improving patient care outcomes?

o   Very effective

o   Effective

o   Neutral

o   Ineffective

o   Very ineffective

18.  Do you believe that digital platforms provide sufficient content variety to meet your learning needs?

o   Strongly agree

o   Agree

o   Neutral

o   Disagree

o   Strongly disagree

19.  What aspects of CME methods do you think require improvement to enhance their effectiveness?

o   Content quality

o   Accessibility

o   Interactive features

o   Duration of modules

o   Others (please specify)

20.  Would you be interested in attending training sessions to improve your digital literacy for CME purposes?

o   Yes

o   No

o   Maybe

Section 6: General Feedback

21. In your opinion, what is the most important factor in choosing a CME method?

o   Flexibility

o   Cost-effectiveness

o   Accessibility

o   Interaction with peers

o   Content quality

22.  Do you have any additional suggestions or comments on improving CME for healthcare professionals?

Appendix 2: Interview questions for CME administrators

Section 1: Background and Context

1. Could you please describe your role and responsibilities as a CME administrator?

2. How long have you been involved in administering CME programs?

3. What types of CME methods have you implemented or overseen during your tenure?

Section 2: Adoption of Innovative CME Methods

4. What are your thoughts on the current shift from traditional CME to more innovative methods, such as microlearning, JITT, and digital platforms?

5. Have you observed an increase in the adoption of microlearning and JITT among healthcare professionals? If yes, what factors do you believe are driving this adoption?

6. How do you perceive the effectiveness of digital platforms compared to traditional in-person CME programs?

Section 3: Barriers and Challenges

7. What are the biggest challenges you have faced in implementing innovative CME methods in your organization?

8. In your experience, what are the main barriers healthcare professionals face when trying to engage with digital CME platforms?

9. Have you encountered resistance from healthcare professionals in adopting these newer CME approaches? If so, what are the reasons behind this resistance?

Section 4: Effectiveness and Engagement

10. In your opinion, how effective are microlearning and JITT in terms of improving healthcare professionals' clinical skills and knowledge retention?

11. How do you ensure that the content provided through digital platforms is engaging and meets the learning needs of healthcare professionals?

12. What feedback have you received from healthcare professionals about the effectiveness of microlearning and JITT in supporting their learning?

Section 5: Digital Literacy and Technological Access

13. How significant is the issue of digital literacy among healthcare professionals when it comes to using digital CME resources?

14. What initiatives, if any, have you put in place to improve digital literacy among healthcare staff?

15. How do technological limitations, such as poor internet access or lack of suitable devices, impact the delivery of CME in your organization?

Section 6: Recommendations and Improvements

16. In your view, what improvements are needed to enhance the effectiveness of innovative CME methods?

17. How do you think healthcare organizations can better support healthcare professionals in adopting these innovative CME methods?

18. What role do you think accreditation and standardization play in the adoption of digital CME platforms?

Section 7: Future Outlook

19. What trends do you foresee in the future of CME, particularly concerning digital and innovative learning methods?

20. How do you envision the role of technology evolving in the field of medical education over the next five years?

Section 8: General Feedback

21. Do you have any additional suggestions or insights on how CME can be made more effective and accessible for healthcare professionals?

22. Is there anything else you would like to add about your experience with CME methods or any challenges/opportunities you see in this field?
